# A global ensemble of ocean wave climate statistics from contemporary wave reanalysis and hindcasts

**DOI:** 10.1038/s41597-022-01459-3

**Published:** 2022-06-22

**Authors:** J. Morim, L. H. Erikson, M. Hemer, I. Young, X. Wang, N. Mori, T. Shimura, J. Stopa, C. Trenham, L. Mentaschi, S. Gulev, V. D. Sharmar, L. Bricheno, J. Wolf, O. Aarnes, J. Perez, J. Bidlot, A. Semedo, B. Reguero, T. Wahl

**Affiliations:** 1 Univeristy of Central Florida (UCF), Orlando, Florida USA; 2 US Geological Survey (USGS), Pacific Coastal Marine Science Center, Santa Cruz, California USA; 3 Commonwealth Scientific and Industrial Research Organisation (CSIRO) Oceans and Atmosphere, Hobart, Tasmania Australia; 4 Department of Infrastructure Engineering, University of Melbourne, Parkville, Victoria Australia; 5 Environment and Climate Change Canada, Climate Research Division, Toronto, Ontario Canada; 6 Disaster Prevention Research Institute, Kyoto University, Kyoto, Japan; 7 Department of Ocean and Resources Engineering, University of Hawai’i at Mānoa, Honolulu, Hawaii USA; 8 European Commission, Joint Research Centre (JRC), Ispra, Italy; 9 Shirshov Institute of Oceanology, Russian Academy of Sciences, Moscow, Russia; 10 National Oceanographic Center (NOC), Liverpool, UK; 11 Geophysical Institute, University of Bergen, Bergen, Norway; 12 MetOcean Solutions, Raglan, New Zealand; 13 European Centre for Medium-range Weather Forecasts (ECMWF), Reading, UK; 14 Department of Water Science and Engineering, IHE-Delft, Delft, The Netherlands; 15 Institute of Marine Sciences, University of California, Santa Cruz, USA

**Keywords:** Physical oceanography, Physical oceanography, Databases

## Abstract

There are numerous global ocean wave reanalysis and hindcast products currently being distributed and used across different scientific fields. However, there is not a consistent dataset that can sample across all existing products based on a standardized framework. Here, we present and describe the first coordinated multi-product ensemble of present-day global wave fields available to date. This dataset, produced through the Coordinated Ocean Wave Climate Project (COWCLIP) phase 2, includes general and extreme statistics of significant wave height (*H*_*s*_), mean wave period (*T*_*m*_) and mean wave direction (*θ*_*m*_) computed across 1980–2014, at different frequency resolutions (monthly, seasonally, and annually). This coordinated global ensemble has been derived from fourteen state-of-the-science global wave products obtained from different atmospheric reanalysis forcing and downscaling methods. This data set has been processed, under a specific framework for consistency and quality, following standard Data Reference Syntax, Directory Structures and Metadata specifications. This new comprehensive dataset provides support to future broad-scale analysis of historical wave climatology and variability as well as coastal risk and vulnerability assessments across offshore and coastal engineering applications.

## Abstract (summary)

**Table:** 

Measurement(s)	Significant wave height • Mean wave period • Mean wave direction
Technology Type(s)	Global wave reanalysis and hindcasts
Sample Characteristic - Environment	Wind-waves
Sample Characteristic - Location	Global

## Background

Wind-generated surface ocean waves have significant environmental^1^, geophysical^2^ and socioeconomic^3^ impacts regionally and globally^4^. It is therefore paramount to understand historical variability and change of wind-wave characteristics over multiple time-scales (monthly, seasonal, and annual)^5^, using high-quality databases with spatial and temporal continuity^6^. To overcome well-reported spatial and temporal limitations of buoy records and satellite radar altimeter measurements^7^, multi-decadal global wind-wave reanalysis and hindcast products have been increasingly used to assess past trends and variability of wave characteristics, particularly significant wave height (*H*_*s*_), mean wave period (*T*_*m*_) and/or mean wave direction (*θ*_*m*_)^7^. These global products have also been widely used to calculate wave-dependent characteristics, such as wave energy, wave setup, and swash^8–10^. These characteristics are commonly used within analysis of long-term historical wave climate change^10^, assessments of historical and future coastal risk^11–13^ considering wind-waves, tides, surges and sea level rise^14,15^ and quantifications of ocean wave energy^16,17^.

In the last decade, numerous multidecadal global wave products have become available. These include the European Centre for Medium-Range Weather Forecasts (ECMWF) set of wave reanalyses (ERA-40^18^, ERA-Interim^19^ and/or ERA5^20^), where wave observations have been assimilated into a coupled atmosphere-wave reanalysis, and a range of wave hindcast products where surface wind fields derived from different global atmospheric reanalyses have been used to force global implementations of spectral global wind-wave models. These products each have different physical wave parameterizations, numerical resolution, data assimilation methods and assimilate different historical observations^21^. As a result, different global wave hindcasts and/or wave reanalyses using different global atmospheric reanalyses as forcing show disparate and often contrasting results regarding climatology, variability, and/or long-term trends^22–25^. These differences are often further complicated and accentuated due to differences among numerical wind-wave modelling methods (e.g., source-term wave parametrizations, numerical resolutions, sea-ice forcing fields and/or bias-correction approaches) available to generate historical wave fields^26^. Despite such differences, most assessments relying on wave characteristics continue to use single pre-selected global wave hindcast or reanalysis products, therefore limiting our confidence in conclusions derived therefrom, as previously discussed^24,27^.

The usage of a single wave product has been often attributed to time and computational constraints as well as suitability since each standalone global wave hindcast or reanalysis has its own temporal resolution and coverage, data format, and accessibility constraints.

This discourages end-users from using a range of wave products. In addition, data quality and contextualization are often an issue as most wave hindcasts or reanalyses have not been intercompared which limits our current understanding^27,28^. Consequently, there is a need for a consistent global multivariate dataset of historical ocean wave fields capable of sampling across different global wave products that is available for widespread use by researchers, stakeholders, private industry and/or governments.

Here, we describe the first community-driven dataset of historical global wave climate assembled from different published global wind-wave hindcast and reanalysis products. This collection assembles a total of fourteen individual global datasets (Supplementary Table 1) and was processed under a pre-established framework developed by the World Meteorological Organization-supported Coordinated Ocean Wave Climate Project (COWCLIP)^29,30^. This global dataset intends to meet current needs from various different perspectives, through the provision of an open-access spatial global wave data collection that lends validated data in consistent format, quality and temporal coverage. The dataset described is archived within Network Common Data Form (netCDF) with CF (Climate and Forecasts) compliant metadata. It provides a variety of standard ocean wave statistics of historical multivariate wave fields (*H*_*s*_, *T*_*m*_, and *θ*_*m*_) over monthly, seasonal and annual time-scales, for 1980 until 2014 (see Supplementary Table 1). The dataset also comprises a new core set of extreme *H*_*s*_ indices advised by the World Climate Research Programme (WCRP)-supported Expert Team on Climate Change Detection (ETCCDI)^31^, providing an additional set of statistics relevant to scientific and engineering applications (Table 2).

This multi-product global ocean wave dataset overcomes several previous limitations, including limited sampling of different wind forcing and wave modelling methods as well as lack of standardization amongst existing global wave hindcasts and reanalysis datasets (e.g., wave variables and their associated statistics and temporal coverage). The purpose is for the dataset to expand as further global-scale wave hindcasts and reanalyses become available. It is expected that open and easy access to such a dataset could, in fact, provide a new stimulus and support assessments of wave climatology, long-term variability and trends, as we look towards improved coastal risk and vulnerability assessments from the climate community^8,32^. It also provides a strong basis for intercomparison analysis with emerging observational wave climate datasets^33^ (e.g. as delivered through ESA’s sea state CCI^34^), or for detection & attribution analysis of uncertainty among products.

## Methods

In this dataset descriptor, we explain the methods and techniques used to generate the original data; the data acquisition processes; the standardized framework employed; the methodology used to generate the vast range of wave parameters and their statistics; and the computational processing used to produce this consistent global dataset. The dataset presented has been compiled from fourteen existing global wave hindcast and reanalysis products, which have been extensively described elsewhere. In this section, we provide a concise description of the original data generated by each wave climate modelling group, with the details of each contribution provided within Supplementary Table 1.

### Global wave hindcasts

#### NCEP/NCAR-driven products

IHC-GOW1.0: Reguero *et al*.^35^ produced the Global Ocean Waves (GOW1.0) wave hindcast by forcing WaveWatch III (hereafter WW3) global wave model version 2.22 with 6-hourly surface wind fields obtained from the NCEP/NCAR atmospheric reanalysis and 1-hourly sea-ice forcing fields from MOM3 sea-ice model. The wave model was implemented using default ST2^36^ source-term physics, with wave spectra discretized over 25 frequencies and 72 directions. The WW3 model was implemented on a global grid with 1.5° × 1.0° spatial resolution with model outputs available at 1-hourly intervals. The GOW1.0 global wave hindcast has undergone a series of calibration and validation methods against significant wave height measurements derived from satellite altimeters and buoy instruments^35^.

#### NCEP CFSR-driven products

CSIRO-G1D: Hemer and Trenham^37^ (hereafter CSIRO-G1D) produced a global wind-wave hindcast using WW3 wave model version 3.14 forced by 1-hourly surface winds from the CFSR atmospheric reanalysis and daily sea-ice forcing fields from MOM4 sea-ice model. The WW3 model was implemented globally at 1° resolution, using ST3 BAJ^36^ source-term physics with the wind-wave growth parameter (*β*_*max*_) adjusted to 1.33. The wave spectra are discretized over 25 frequencies and 24 directions and the model outputs are available at 1-hourly intervals. CSIRO-G1D has been compared against ECMWF’s ERA-Interim and ERA-40C using a range of skill metrics.

CSIRO-CAWCR: Smith *et al*.^38^ (hereafter CSIRO-CAWCR) presented a global wave hindcast using versions 4.08/v4.18 of WW3. The atmospheric forcing of WW3 were hourly surface winds derived from CFSR atmospheric reanalysis over 1979–2015. Sea-ice concentration fields at hourly intervals from MOM4 sea-ice model were used as forcing. The wave model was setup at 0.4° resolution using ST4^36^ source-term physics using default settings. The wave spectra are discretized across 29 frequencies and 24 directions, with model outputs available at 1-hourly resolution.

IHC-GOW2.0: Perez *et al*.^39^ (hereafter IHC-GOW2.0) produced an updated global hindcast of GOW1.0^30^ driven by hourly surface wind fields from CFSR atmospheric reanalysis and hourly sea-ice forcing from MOM4 sea-ice model. The GOW2.0 is based on version 4.18 of WW3 and uses default ST4^36^ source-term physics package. The model was implemented on a multi-grid scheme with a series of two-way nested domains covering global oceanic basins at ~0.5° spatial resolution and continental shelf areas at ~0.25° spatial resolution. The wave spectra are discretized over 32 frequencies and 24 directions and outputs are available at 1-hourly intervals. The model data has been validated against wave spectral information from buoy stations and multi-mission satellite altimeter measurements^39^.

JRC-CFSR: Mentaschi *et al*.^40^ (hereafter JRC-CFSR) developed a global wave hindcast by forcing WW3 wave model version 4.08 with near-surface wind fields from CFSR global atmospheric reanalysis. The WW3 model was implemented without sea-ice forcing. The model setup uses ST4^36^ source-term physics the wave growth parameter (*β*_*max*_) adjusted to 1.52. The model domain consists of a global grid at 1.5° spatial resolution, with nested sub-grids implemented across specific regions at 0.25 and 0.5° spatial resolutions. Model outputs are available at 3-hourly resolution. JRC-CFSR has been compared against multi-mission satellite-retrieved measurements, buoy observations and global wave hindcast - JRC-ERAI^30^.

IFREMER-CFSRMOD: Stopa *et al*.^41^ (hereafter IFREMER-CFSRMOD) created a global wave hindcast by forcing WW3 model version 5.16 with satellite-adjusted hourly surface winds from CFSR atmospheric reanalysis and hourly sea-ice forcing obtained from MOM4 sea-ice model. The model was setup using ST4^36^ source-term physics with *β*_*max*_ adjusted to 1.30 and wave spectra discretized over 24 frequencies and 32 directions. The model wave outputs are archived at 1-hourly resolution at 0.5° spatial resolution. This hindcast has been compared against buoy observations and satellite altimeter measured data^41^.

#### ECMWF ERAI-driven products

JRC-ERAI: Mentaschi *et al*.^40^ (hereafter JRC-ERAI) generated a global wind-wave hindcast by forcing WW3 wave model version 4.08 with 6-hourly surface wind from ECMWF ERA-Interim atmospheric reanalysis. The WW3 model was run without sea-ice forcing using the ST4^36^ source-term physics with default settings. The model was implemented at 1° spatial resolution with outputs available at 12-hourly intervals.

NOC-ERAI: Bricheno and Wolf^42^ (hereafter NOC-ERAI) developed a global wave hindcast using WW3 wave model version 3.14, forced by 6-hourly surface wind fields derived from ECMEF ERA-Interim atmospheric reanalysis and daily sea-ice concentrations from LIM2 sea-ice model. The model was implemented using default ST2^36^ source-term physics with wave spectra discretized across 30 frequencies and 36 directions. The spatial resolution was set at ~0.7° × 0.5° with outputs available at 1-hourly intervals.

#### ECMWF ERA5-driven products

ECMWF-ERA5H: ECMWF-ERA5H: Bidlot *et al*.^43,44^ (hereafter ECMWF-ERA5H) created a global wave hindcast by forcing EC-WAM wave model with 1-hourly atmospheric forcing and sea ice cover from ECMWF ERA5 atmospheric reanalysis. The EC-WAM wave model was implemented at 0.5° spatial resolution, with spectral ordinates discretized over 36 frequencies and 36 directions. The model settings included ST4 source-term physics^36^ tuned to ECMWF Earth System model^45^. ECMWF-ERA5H surface wave parameters have been compared against both satellite altimeter measurements and buoy observations^43^.

#### JMA JRA-55-driven products

KU-JRA-55-ST2: Mori *et al*.^46,47^ (hereafter KU-JRA-55ST4) created a global wave hindcast by driving the WW3^36^ wave model version 4.18 using 6-hourly surface wind fields from JRA-55 atmospheric reanalysis and monthly sea-ice concentration fields from COBE sea-ice model. The WW3 model was implemented using default ST2^36^ source-term physics with wave spectra discretized over 29 frequencies and 30 directions. The model domain consists of a global grid with 0.56° resolution and model outputs are archived at 1-hourly intervals.

KU-JRA-55ST4: Shimura *et al*.^46,47^ (hereafter KU-JRA55-ST4) created a global ocean wave hindcast by forcing WW3 wave model version 4.18 with 6-hourly surface wind fields from JRA-55 atmospheric reanalysis and monthly sea-ice concentration fields from COBE sea-ice model. The wave model was implemented using the default ST4^36^ source-term physics with spectra discretized over 29 frequencies and 30 directions. The domain consists of a global grid with 0.56° spatial resolution and the model outputs are archived at 1-hourly intervals.

#### NASA MERRA2-driven products

IORAS-MERRA2: Sharmar *et al*.^24^ (hereafter IORAS-MERRA2) created a global wind-wave hindcast by forcing WW3 wave model version 5.03 with 6-hourly surface winds obtained from NASA GMAO MERRA2 atmospheric reanalysis. Hourly sea-ice concentration fields were taken from the MERRA2 coupled sea-ice model. The model was implemented using ST4^36^ source-term physics with default settings, with model outputs archived on a global grid with 0.5° × 0.625° spatial resolution at 6-hourly intervals. The IORAS-MERRA2 global wave hindcast has been compared against other wave hindcasts, visual observations and satellite altimeter measurements^24^.

### Global wave reanalyses

ECMWF-ERAI: Dee *et al*.^19^ (hereafter ECMWF-ERAI) generated the fourth generation of ECMWF´s atmospheric reanalysis by combining model data with historical observations.

ECMWF-ERAI was produced using a 4D-VAR data assimilation system as part of ECMWF Integrated Forecasting System (IFS) CY31R2^45^ and provides 6-hourly atmospheric fields at 0.70° spatial resolution from 1979-onwards. The ocean wave parameters are available 3-hourly at 1.5° spatial resolution and are derived from a fully-coupled atmosphere-wave model (WAM^45^) that describes the time-evolution of ocean wave spectra with assimilated satellite-retrieved wave height data from 1991 onwards to adjust model-simulated wave spectra based on assumptions about contributions of wind-sea and swells. ECMWF-ERAI wave parameters have been compared against satellite altimetry and buoy records^19,23^.

ECMWF-ERA5: Hersbach *et al*.^20^ (hereafter ECMWF-ERA5) developed the fifth generation of ECMWF atmospheric reanalysis which combines model data with vast amounts of past re-processed observations from across the world into a globally complete and consistent dataset. The ECMWF-ERA5 was designed using a 4D-VAR sophisticated data assimilation method as part of ECMWF Integrated Forecasting System (IFS) CY41R2^45^ and provides hourly atmospheric fields at 0.25° spatial resolution from 1979-onwards. The ocean wave parameters are generated from a fully-coupled atmosphere-wave model (WAM^45^) using assimilated satellite radar altimeter derived wave height data (from 1991-onwards). The model comprises various enhancements over its previous versions, with 1-hourly model outputs available at 0.5° spatial resolution.

### Data processing framework

**Fig. 1:**
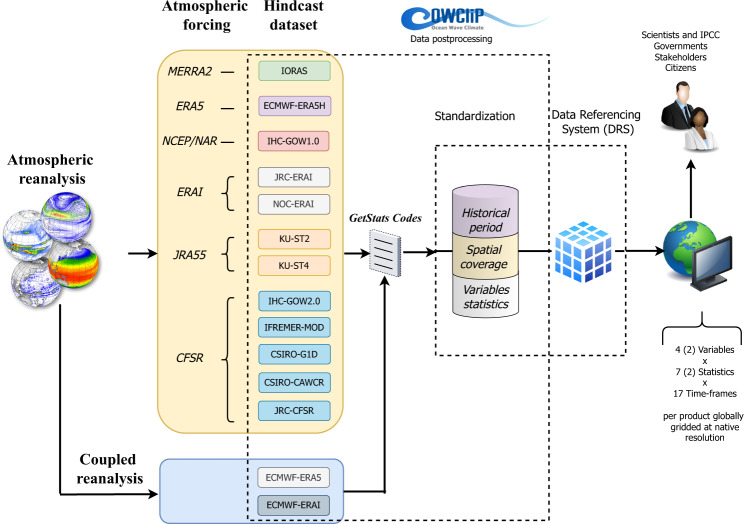
Flowchart of the experimental framework used.

Whereas each of the global ocean wind-wave products were developed independently, a working protocol was defined to provide a systematic, community-driven framework and infrastructure to support validation, intercomparison, documentation and access for historical global wave hindcasts or reanalyses. Based on this framework, we focus on a set of integrated wave parameters (*H*_*s*_, *T*_*m*_ and *θ*_*m*_) from which a set of standard statistics were obtained (at annual, seasonal and monthly time-frame resolutions) in a consistent manner (Tables 1, 2) as shown below in the *Data Generation Method* section. The resulting data across three frequencies and three variables, capturing seven statistical measures (for *H*_*s*_ and *T*_*m*_ and two for *θ*_*m*_) and seven annual extremes statistics represent the entire dataset of historical global wave products. The flowchart of the COWCLIP experimental framework used is illustrated in Fig. 1.

**Table 1:** Summary of the wave variables and standard statistics included within the dataset.

Variable	Statistics ID	Indicator name	Time-frame resolutions	Units
getStat.f				
*H*_*s*_	*H*_*s*__avg	Mean significant wave height	Annual (1), Seasonal (4) and Monthly (12)	m
	*H*_*s*__p10	10th Percentile significant wave height	Annual (1), Seasonal (4) and Monthly (12)	m
	*H*_*s*__p50	50th Percentile significant wave height	Annual (1), Seasonal (4) and Monthly (12)	m
	*H*_*s*__p90	90th Percentile significant wave height	Annual (1), Seasonal (4) and Monthly (12)	m
	*H*_*s*__p95	95th Percentile significant wave height	Annual (1), Seasonal (4) and Monthly (12)	m
	*H*_*s*__p99	99th Percentile significant wave height	Annual (1), Seasonal (4) and Monthly (12)	m
	*H*_*s*__max	Maximum significant wave height	Annual (1), Seasonal (4) and Monthly (12)	m
*T*_*m*_^a^	*T*_*m*__avg	Average mean wave period	Annual (1), Seasonal (4) and Monthly (12)	s
	*T*_*m*__p10	10th Percentile mean wave period	Annual (1), Seasonal (4) and Monthly (12)	s
	*T*_*m*__p50	50th Percentile mean wave period	Annual (1), Seasonal (4) and Monthly (12)	s
	*T*_*m*__p90	90th Percentile mean wave period	Annual (1), Seasonal (4) and Monthly (12)	s
	*T*_*m*__p95	95th Percentile mean wave period	Annual (1), Seasonal (4) and Monthly (12)	s
	*T*_*m*__p99	99th Percentile mean wave period	Annual (1), Seasonal (4) and Monthly (12)	s
	*T*_*m*__max	Maximum mean wave period	Annual (1), Seasonal (4) and Monthly (12)	s
getStatDir.f				
*θ*_*m*_ (dir)	*θ*_*m*__avg	Circular mean	Annual (1), Seasonal (4) and Monthly (12)	°N
	*θ*_*m*__std	Circular standard deviation	Annual (1), Seasonal (4) and Monthly (12)	°N

^a^Mean wave period using spectral moments of order 0 and 1 unless specified otherwise.

**Table 2:** Summary of the ETCCDI set of extreme significant wave height statistics included within the dataset.

ETCCDI set of *H*_*s*_ statistics			
Statistics ID	Indicator name	Definition	Units
getstaHsEx.f			
HsRo	Rough wave days	Annual count of days when daily max *H*_*s*_ > 2.5 m	days
HsHi	High wave days	Annual count of days when daily max *H*_*s*_ > 6 m	days
fHsRo	Frequency of rough wave days	Annual percentage of days when daily max *H*_*s*_ > 2.5 m	%
fHsHi	Frequency of high wave days	Annual percentage of days when daily max *H*_*s*_ > 6 m	%
fHs10p^a^	Frequency of low decile wave days	Annual percentage of days when daily max *H*_*s*_ < 10th percentile of daily max *H*_*s*_ in the base period^a^	%
fHs90p^a^	Frequency of top decile wave days	Annual percentage of days when daily max *H*_*s*_ > 90th percentile of daily max *H*_*s*_ in the base period^a^	%
HHsDI^a^	Top decile wave spell duration indicator	Annual count of days with at least 2 consecutive days when daily max *H*_*s*_> 90th percentile of daily max *H*_*s*_ in the base period^a^	day

^a^Relative statistics with base period 1980–2005 used for bootstrap procedure in relative statistics.

### Data generation method

As part of the COWCLIP community framework, a set of codes were developed to ensure a consistent computational data processing. The codes contain three functions (getStat.f, getStatDir.f, getHsEx.f) that are used to calculate two standard sets of statistics, using sub-daily raw data from each standalone dataset. During processing, the data were written to netCDF4 format. For information on access to and guidelines for setup/usage of COWCLIP codes, see the Code Availability section.

#### Standard statistics - getStat.f and getStatDir.f

The getStat.f code was designed to estimate statistics valid for scalar variables (*H*_*s*_, *T*_*m*_). The code was applied to each individual wave dataset separately, enabling the calculation of seven wave climate statistics (Mean, 10th, 50th, 90th, 95th, 99th percentiles as well as maximum values) for *H*_*s*_ and *T*_*m*_ calculated over monthly, seasonal and annual time-frame resolutions. Seasonal statistics were computed across four default seasons defined as DJF (December to February), MAM (March to May), JJA (July to August) and SON (September to November). The output netCDF files derived from each specific dataset retained all the relevant metadata of the input file and the coordinate variables and statistics. The names of the output files contained the time-frames of the statistics processed and the temporal resolution of the input data.

The getStatDir.f code is analogous to getStat.f, but it was designed to calculate circular statistics meaningful for directional variables (such as *θ*_*m*_). The code was applied to each standalone dataset (with available *θ*_*m*_) providing 2 circular statistics (mean and standard deviation) at monthly, seasonal and annual time-frames (Table 1).

#### Extreme statistics - getHsEx.f

The getHsEx.f code was designed to calculate an ETCCDI set of extreme annual *H*_*s*_ indices from the sub-daily *H*_*s*_ input data (see Table 2). The code was applied to each independent dataset separately after concatenating all the standard historical data. A defined baseline period across 1986–2005 for relative statistics was used following the community-based framework^29,30^. The output netCDF files contain seven extreme wave statistics calculated annually.

### Data assembly method

The netCDF files derived from each standalone dataset using the code described above, were used as a basis to build this collection of historical global wave products following the standardization framework (Fig. 1). Before assembling, each independent netCDF file was quality-controlled. The relevant ocean wave statistics were extracted from each file, obtained from each standalone wave dataset. Given the broad range of spatial resolutions amongst products (Supplementary Table 1), and to give the users access to the original resolution of the global wave datasets, no interpolation method was used. Subsequently, the annual, seasonal and monthly statistics across the common time period amongst the wave datasets (between 1980–2014) were selected for further processing. We note that some specific products (KU-JRA55-ST2, KU-JRA55-ST4, or CSIRO-G1D) do not extend to 2014 (Table 3); however, we still processed and compiled their data since users might be interested in using the full multi-product ensemble data at shorter temporal windows. The resultant wave data are thus consistent in terms of wave variables, their general and extreme wave statistics, and temporal coverage, without ‘undesirable’ inconsistencies (which have previously limited intercomparison assessments). The data produced were controlled to be unchanged between the different formats throughout the process.

**Table 3:** Time-frames available for each global wave product included within the dataset.

Name of product	Time period	Starting Year (ANL)	Starting Season (SNL)	Starting Month (MLY)
ECMWF-ERA5	1980–2014	1980	DJF	January
ECMWF-ERAI	1980–2014	1980	DJF	January
IHC-GOW1.0	1980–2014	1980	DJF	January
ECMWF-ERA5H	1980–2014	1980	DJF	January
KU-JRA-55-ST2	1980–2004	1980	DJF	January
KU-JRA-55-ST4	1980–2012^b^	1980	DJF	January
IORAS-MERRA2	1980–2014	1980	MAM^b^	January
NOC-ERAI	1980–2014	1980	DJF	January
IFREMER-CFSRMOD	1980–2014	1980	MAM^b^	January
IHC-GOW2.0	1980–2014	1980	DJF	January
CSIRO-G1D	1980–2010^b^	1980	DJF	January
CSIRO-CAWCR	1980–2014	1980	DJF	January
JRC-CFSR	1980–2014	1980	MAM^b^	March^b^
JRC-ERAI	1980–2014	1980	MAM^b^	January

^a^Any missing seasons and/or months have been populated with missing values.

## Data Records

The full global archived dataset^48^ comprising the different statistics described (see Data Generation Method) can be accessed via a Scientific Data recommended data repository: Australian Ocean Data Network (AODN) at 10.26198/3kkc-2g71.

The data set in total comprises 134 files. The data is structured with a consistent directory structure and file naming conventions following our COWCLIP2.0 dataset^49^ (and akin to that used in CMIP projects) when possible for consistency:

                                                                                             Directories

                                                                    hindcasts/<frequency>/<variable>/

                                                                                             Filenames

                         <variable>_<modelling_centre>_<frequency>_<start_date>-<end_date>.nc

The wave data were made CF compliant by ensuring the ‘standard_name’ field was not erroneously used and the variable ‘long_name’ was defined consistent with the code and units applied. No value for ‘_FillValue’ was provided and therefore this has been omitted. Recommended global attributes are defined and included, drawing from the COWCLIP metadata table (see Supplementary Table 1) which enables additional compliance with the ACDD metadata standard.

Note that although every effort was made so as to ensure data adhered to both the CF and ACDD metadata conventions, the files are not strictly CF-compliant in time dimension - which uses units “years since” and “months since” the reference date. This is not advised by the CF convention since these values are ambiguous and depend on the calendar used. As input data comes from different global wave products and groups which use a variety of calendars and this information is not captured within the data generated by the getStat scripts, retrospectively applying calendar definitions was deemed to be less appropriate than using the more generic time definition - which is in line with the data produced by getStat.

## Technical Validation

All contributing products have been assessed for model skill against buoy observations, satellite altimetry records and/or specific global wave hindcast or global wave reanalysis datasets^23,24,35,38,39^. Intercomparison of wave model skill in terms of mean and extreme significant wave height climatology and against an extensively calibrated, cross-validated reference global satellite dataset called IMOS^33^ have also been extensively conducted.

## Supplementary information

Supplementary Materials

## Data Availability

Fortran code: getStat.f, getStatDir.f, getHsEx.f. The Fortran code developed to calculate the COWCLIP statistics can be requested via the COWCLIP website (https://cowclip.org/data-access). The code - as described in the Data Generation Method section, consists of a set of code commands (getStat.f, getStatDir.f and getHsEx.f) which can be compiled with a Fortran compiler, linked against netCDF4 and HDF5 libraries. The documentation for setup, usage and requirements for the code is described within the technical reports^29,30^ which complement this data descriptor. These commands can be executed by COWCLIP contributors to generate the set of ocean wave statistics from their raw simulations. With the specific purpose of sharing in an open data format, and adhering to relevant data standards, the processed data is given in netCDF format, the global metadata attributes from the submitted netCDF data recorded, and additional information added where possible to ensure both CF Conventions & Attribute Convention for Dataset Discovery (‘ACDD’) standards compliance.
