# Author Correction: A global ensemble of ocean wave climate statistics from contemporary wave reanalysis and hindcasts

**DOI:** 10.1038/s41597-022-01519-8

**Published:** 2022-07-08

**Authors:** J. Morim, L. H. Erikson, M. Hemer, I. Young, X. Wang, N. Mori, T. Shimura, J. Stopa, C. Trenham, L. Mentaschi, S. Gulev, V. D. Sharmar, L. Bricheno, J. Wolf, O. Aarnes, J. Perez, J. Bidlot, A. Semedo, B. Reguero, T. Wahl

**Affiliations:** 1 Univeristy of Central Florida (UCF), Orlando, Florida USA; 2 US Geological Survey (USGS), Pacific Coastal Marine Science Center, Santa Cruz, California USA; 3 Commonwealth Scientific and Industrial Research Organisation (CSIRO) Oceans and Atmosphere, Hobart, Tasmania Australia; 4 Department of Infrastructure Engineering, University of Melbourne, Parkville, Victoria Australia; 5 Environment and Climate Change Canada, Climate Research Division, Toronto, Ontario Canada; 6 Disaster Prevention Research Institute, Kyoto University, Kyoto, Japan; 7 Department of Ocean and Resources Engineering, University of Hawai’i at Mānoa, Honolulu, Hawaii USA; 8 European Commission, Joint Research Centre (JRC), Ispra, Italy; 9 Shirshov Institute of Oceanology, Russian Academy of Sciences, Moscow, Russia; 10 National Oceanographic Center (NOC), Liverpool, UK; 11 Geophysical Institute, University of Bergen, Bergen, Norway; 12 MetOcean Solutions, Raglan, New Zealand; 13 European Centre for Medium-range Weather Forecasts (ECMWF), Reading, UK; 14 Department of Water Science and Engineering, IHE-Delft, Delft, The Netherlands; 15 Institute of Marine Sciences, University of California, Santa Cruz, USA

**Keywords:** Physical oceanography, Physical oceanography, Databases, Physical oceanography, Physical oceanography, Databases

Correction to: *Scientific Data* 10.1038/s41597-022-01459-3, published online 22 June 2022

In this article the affiliation details for Claire Trenham were incorrectly given as ‘US Geological Survey (USGS), Pacific Coastal Marine Science Center, Santa Cruz, California, US’ but should have been ‘Commonwealth Scientific and Industrial Research Organisation (CSIRO) Oceans and Atmosphere, Hobart, Tasmania, Australia ‘. The original article has been corrected.

