# Supplementary material for: A global ensemble of ocean wave climate statistics from contemporary wave reanalysis and hindcasts
Source: Sci Data. 2022 Jun 22;9:358. doi: 10.1038/s41597-022-01459-3 (PMC9217809; doi:10.1038/s41597-022-01459-3)
Supplement: Supplementary file 1 — Supplementary Materials [file 41597_2022_1459_MOESM1_ESM.docx]

**A global ensemble of ocean wave climate statistics from contemporary wave reanalysis and hindcasts**

Morim, J.^1^, Erikson, L.H.^2^, Hemer, M., Young, I.^4^., Wang, X.^5^, Mori, N.^6^, Shimura, T.^6^, Stopa, J.^7^, Trenham, C.^2^, Mentaschi, L.^8^, Gulev, S.^9^, Sharmar, V.D.^9^, Bricheno, L.^10^, Wolf, J.^10^, Aarnes, O.^11^, Perez, J.^12^, Bidlot, J.^13^, Semedo, A.^14^, Reguero, B.^15^, Wahl, T.^1^,

^1^Univeristy of Central Florida (UCF), Orlando, Florida US.

^2^US Geological Survey (USGS), Pacific Coastal Marine Science Center, Santa Cruz, California, US.

^3^Commonwealth Scientific and Industrial Research Organisation (CSIRO) Oceans and Atmosphere, Hobart, Tasmania, Australia.

^4^Department of Infrastructure Engineering, University of Melbourne, Parkville, Victoria, Australia.

^5^Environment and Climate Change Canada, Climate Research Division, Toronto, Ontario, Canada.

^6^Disaster Prevention Research Institute, Kyoto University, Kyoto, Japan.

^7^Department of Ocean and Resources Engineering, University of Hawaiʻi at Mānoa, Honolulu, Hawaii, US.

^8^European Commission, Joint Research Centre (JRC), Ispra, Italy

^9^Shirshov Institute of Oceanology, Russian Academy of Sciences, Moscow, Russia

^10^National Oceanographic Center (NOC), Liverpool, UK.

^11^Geophysical Institute, University of Bergen, Bergen, Norway.

^12^MetOcean Solutions, Raglan, New Zealand.

^13^European Centre for Medium-range Weather Forecasts (ECMWF), Reading, UK.

^14^Department of Water Science and Engineering, IHE-Delft, Delft, The Netherlands.

^15^Institute of Marine Sciences, University of California, Santa Cruz, US.

*Corresponding author address:

Dr. Joao Morim

Department of Civil, Environmental and Construction Engineering, University of Central Florida,

Orlando, FL, United States

Email: jmorimnascimento@ucf.edu

| **Table 1** – Summary of the global wave contributions to the ensemble dataset. | | | | | | | | | | | |  | |  | |  |  |
| --- | --- | --- | --- | --- | --- | --- | --- | --- | --- | --- | --- | --- | --- | --- | --- | --- | --- |
| Name of product | Atmospheric Reanalysis^e^  (Levels) | Assimilation System and Method | Spatial and Temporal Resolution | Sea-Ice Model | Wave Model | Input/  dissipation source-terms | Spatial and Temporal Resolution | Spectral Resolution | Bathymetry Source | Bias  Adjust. | Wave Variables | |  | |  | | |
| ECMWF-ERA5 | ERA5  (L137) | IFS 41R2/  4D-VAR | 0.25° / 1h | OSI-SAF (daily) | EC-WAM  (CY41R2) | ST3 (default) | 0.36°/ 1h | 30f x 24d | ETOPO2 | None | $H_{s}$,$T_{m}, \theta_{m}$ | | Reanalysis | | | |  |
| ECMWF-ERAI | ERA-Interim  (L91) | IFS 31R2/  4D-VAR | 0.75° / 6h | OSI-SAF (daily) | EC-WAM  (CY31R2) | ST3 (default) | 1.0° / 6h | 30f x 24d | ETOPO2 | None | $H_{s}$,$T_{m}, \theta_{m}$ | |  |  |  |  |  |
| IHC-GOW1.0 | NCEP/NCAR  (L28) | GDAS/  3D-VAR | 1.90° x 1.87° / 6h | MOM3  (hourly) | WWIII v2.22 | ST2 (default) | 1.50° x 1.0° / 6h | 25f x 72d | ETOPO2 | Altimetry $H_{s}$ | $H_{s}$,$T_{m}, \theta_{m}$ | | Reanalysis-driven hindcasts | | | |  |
| ECMWF-ERA5H | ERA5  (L137) | IFS 41R2/  4D-VAR | 0.25° / 1h | ERA5 (daily) | EC-WAM  (CY46R1) | ST4 (default) | 0.50° / 1h | 36f x 36d | ETOPO1 | None | $H_{s}$,$T_{m}, \theta_{m}$ | |  |  |  |  |  |
| KU-JRA-55-ST4 | JRA55  (L60) | GSM-  4D-VAR | 0.56° / 6h | COBE (monthly) | WWIII v4.18 | ST4 (default) | 0.56° / 1h | 29f x 30d | ETOPO5 | None | $H_{s}$,$T_{m}, \theta_{m}$ | |  |  |  |  |  |
| KU-JRA-55-ST2 | JRA55  (L60) | GSM-  4D-VAR | 0.56° / 6h | COBE (monthly) | WWIII v4.18 | ST2 (default) | 0.56° / 1h | 29f x 30d | ETOPO5 | None | $H_{s}$,$T_{m}, \theta_{m}$ | |  |  |  |  |  |
| IORAS-MERRA2 | MERRA2  (L72) | GEOS-5-  3D-VAR | 0.5° x 0.62° /  6h | MERRA2  (hourly) | WW3  V5.03 | ST4 (default) | 0.50 x 0.62° / 6h | 32f x 24d | ETOPO2 | None | $H_{s}, \theta_{m}$ | |  |  |  |  |  |
| NOC-ERAI | ERA-Interim  (L91) | 4D-VAR (R1) | 0.75° / 6h | LIM2 (daily) | WWIII v3.14 | ST2 (default) | 0.70 x 0.47° / 1h | 30f x 36d | ETOPO2 | None | $H_{s}, \theta_{m}$ | |  |  |  |  |  |
| IFREMER-CFSRMOD | CFSR  (L64) | GDAS/CFS-3D-VAR | 0.50° / 1h | MOM4  (hourly) | WWIII v5.16 | ST4  ($\beta$^b^ = 1.30) | 0.50° / 1h | 32f x 24d | ETOPO5 | Altimetry $U_{10}$ | $H_{s}$,$T_{m}$^a^ | |  |  |  |  |  |
| IHC-GOW2.0 | CFSR  (L64) | GDAS/CFS-3D-VAR | 0.50° / 1h | MOM4  (hourly) | WWIII v4.18 | ST4  ($\beta$^b^ = 1.26) | 0.25-0.50°  / 1h | 32f x 24d | ETOPO1 | None | $H_{s}$,$T_{m}, \theta_{m}$ | |  |  |  |  |  |
| CSIRO-G1D | CFSR  (L64) | GDAS/CFS-3D-VAR | 0.50° / 1h | MOM4  (daily) | WWIII v3.14 | ST3 BAJ (default) | 1.0° / 1h | 25f x 24d | DBDB2v3 | None | $H_{s}$,$T_{m}, \theta_{m}$ | |  |  |  |  |  |
| CSIRO-CAWCR | CFSR  (L64) | GDAS/CFS-3D-VAR | 0.50° / 1h | MOM4  (hourly) | WWIII v3.14 | ST4  ($\beta$^b^ = 1.33) | 0.40° / 1h | 29f x 24d | DBDB2v3 | None | $H_{s}$,$T_{m}, \theta_{m}$ | |  |  |  |  |  |
| JRC-CFSR | CFSR  (L64) | GDAS/CFS-3DVAR | 0.50° / 1h | None | WW3 | ST4  ($\beta$^b^ = 1.52) | 1.5-0.50°  / 3h | 25f x 24d | ETOPO5 | None | $H_{s}$,$T_{m}, \theta_{m}$ | |  |  |  |  |  |
| JRC-ERAI | ERA-Interim  (L91) | 4D-VAR (R2)^g^ | 0.75° / 6h | None | WW3 | ST4 (default) | 1.0° / 12h | 25f x 24d | ETOPO5 | None | $H_{s}$,$T_{m}, \theta_{m}$ | |  | | | |  |

^a^mean wave period using spectral moments of order 0 and 2.

^b^$\beta$ is the wind-wave growth term.
